# Building a super elongation complex for HIV

**DOI:** 10.7554/eLife.00577

**Published:** 2013-03-08

**Authors:** Christopher P Hill, Wesley I Sundquist

**Affiliations:** 1 **Christopher P Hill** is at the Department of Biochemistry, University of Utah School of Medicine, Salt Lake City, United Stateschris@biochem.utah.edu; 2 **Wesley I Sundquist** is an *eLife* reviewing editor, and is at the Department of Biochemistry, University of Utah School of Medicine, Salt Lake City, United Stateswes@biochem.utah.edu

**Keywords:** transcription elongation, super elongation complex, SEC, intrinsically disordered proteins, structural biology, Human

## Abstract

A better understanding of the host cell protein complex that helps HIV replicate inside cells offers the possibility of new therapeutic targets.

**Related research article** Schulze-Gahmen U, Upton H, Birnberg A, Bao K, Chou S, Krogan NJ, Zhou Q, Alber T. 2013. The AFF4 scaffold binds human P-TEFb adjacent to HIV Tat. *eLife* **2**:e00327. doi: 10.7554/eLife.00327

**Image** Model of part of the HIV replication complex

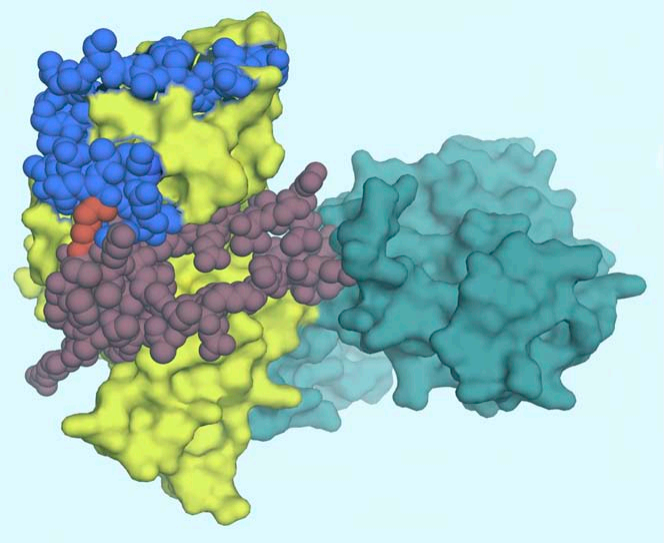

AIDS, which is estimated to have claimed the lives of more than 30 million people worldwide, is caused by HIV, a member of the lentivirus family of single-stranded RNA viruses. HIV infects cells that belong to the immune system; when the virus enters a cell, a viral enzyme converts HIV RNA into double-stranded DNA through a process called reverse transcription. The viral DNA then moves to the nucleus, where another viral enzyme integrates it into the host cell's own DNA. From this point onwards, the virus can either remain latent (and invisible to the host immune system) or it can begin to replicate to produce more virus particles. To produce its genetic material, HIV ‘hijacks' the cell's gene expression machinery, forcing a cellular enzyme called RNA polymerase II to transcribe viral DNA along with the cell's own DNA.

The HIV genome encodes a protein called Tat that promotes the transcription of viral DNA (Ott et al., 2011; Razooky and Weinberger, 2011). In the absence of Tat, RNA polymerase II begins to transcribe viral DNA into RNA, but typically pauses after copying fewer than 50 nucleotides. Tat stimulates HIV transcription by binding to newly formed viral transcripts at a hairpin-shaped—or stem-loop—structure called TAR. The Tat:TAR complex then binds an enzyme called positive transcription elongation factor b (P-TEFb), which itself is a complex of two proteins, CDK9 and cyclin T1. P-TEFb releases the paused RNA polymerase II by phosphorylating cellular proteins that would otherwise inhibit transcriptional elongation, and the polymerase tail, which is then able to recruit proteins important for elongation. P-TEFb also promotes the binding of additional elongation factors to form an assembly called the ‘super elongation complex', which is organized by highly flexible scaffolding proteins, such as AFF4 (Figure 1) (He et al., 2010; Sobhian et al., 2010; Luo et al., 2012; Chou et al., 2013).

**Figure 1.**
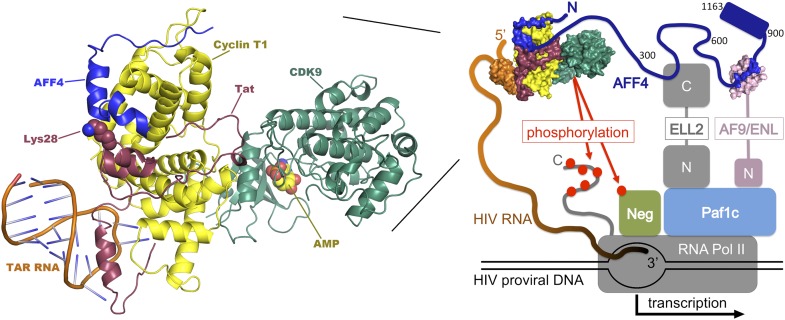
Transcription of DNA in a cell infected by HIV. The HIV DNA that has been integrated into the host cell's genome is transcribed by the cellular enzyme RNA polymerase II (RNA Pol II), but this process usually pauses after fewer than 50 nucleotides have been transcribed. In order to synthesize long transcripts, the HIV genome encodes a protein called Tat (shown here in maroon), which binds newly formed viral RNA transcripts (orange) at a hairpin-shaped structure called TAR. The Tat:TAR complex then binds a protein complex called P-TEFb (which is composed of cyclin T1 [yellow] and CDK9 [green]), plus a scaffolding protein, AFF4 (blue; numbers correspond to amino-acid positions within the protein). Note that Tat and AFF4 may interact directly on the cyclin T1 surface and that acetylation of a lysine residue at position 28 in Tat (which has been shown to enhance Tat activity; Ott et al., 2011) may help regulate this interaction. The AFF4 tethers additional elongation factors (ELL-2 and either ENL or AF9; only shown in the right-hand figure, ‘N' and ‘C' represent the N and C terminal domains) to produce a ‘super elongation complex' that regulates the transition to efficient transcriptional elongation. Left, expanded model of the P-TEFb:AFF4:Tat:TAR complex. Right, overview of the interactions within the super elongation complex, including interactions with RNA Pol II and the Paf1 complex (Paf1c), which is a positive regulator of transcription. Phosphorylation (red dots) by P-TEFb of negative factors that inhibit transcription (NELF and DSIF, abbreviated ‘Neg') and of the C-terminal tail of RNA Pol II, releases the stalled polymerase and increases transcription of the viral genome. The composite model shown here was assembled from structures of human Cdk9:cyclin T1:AFF4 (pdb 4imy), Tat:p-TEFb (pdb 3mi9), and EIAV Tat:TAR (pdb 2w2h).

Now, writing in *eLife*, Tom Alber of the University of California at Berkeley and colleagues—including Ursula Schulze-Gahmen as first author—report a crystal structure that reveals the molecular basis for the interactions between P-TEFb and AFF4, helps to reveal how this complex is bound by the viral Tat protein, and suggests new strategies for therapeutic intervention (Schulze-Gahmen et al., 2013). The crystal structure reveals that part of the polypeptide chain of the AFF4 scaffolding protein (specifically residues 34–66) snakes across the surface of cyclin T1, some distance from CDK9 (Figure 1). The AFF4 polypeptide conformation lacks contacts between regions of AFF4 that are separated by more than a few residues in the amino acid sequence, consistent with the idea that AFF4 becomes well-ordered only upon P-TEFb binding (Chou et al., 2013). Residues in cyclin T1 shift somewhat to accommodate the AFF4 polypeptide, and the interaction between the two proteins is stabilized by a series of hydrophobic contacts and eight inter-molecular hydrogen bonds.

Alber and co-workers report three pieces of evidence to support their structure. First, they show that an AFF4 fragment spanning only residues 33–67 binds cyclin T1 with nearly full affinity (although there is some indication that AFF4 residues at the N-terminal end of the molecule may also make weak contacts with CDK9). Second, they show that mutation of cyclin T1 surface residues that contact AFF4 impairs binding. And third, they show that mutation of AFF4 residues that contact cyclin T1 reduces transcription from the HIV promoter.

Unexpectedly, the new crystal structure suggests how HIV Tat could recruit the super elongation complex. In conjunction with a previously reported Tat-cyclin T1 structure (Tahirov et al., 2010), Alber and colleagues show that AFF4 and Tat bind adjacent to each other on cyclin T1. They also show that AFF4 and Tat bind synergistically to P-TEFb, and that functionally important Tat residues are well positioned to contact AFF4. Thus, consistent with functional data (D'Orso et al., 2012), it appears that AFF4 and Tat interact directly when bound on the surface of P-TEFb, although details of this interaction have yet to be structurally characterized.

The new P-TEFb:AFF4 structure touches on a number of intriguing questions that merit further study. For example, how do other components of the super elongation complex interact with their distinct binding sites along the extended AFF4 scaffold? And do the different elongation factors interact with one another when held in close proximity? To this end, a recent analysis of the interaction between the binding domain of the elongation factor AF9 and its binding site on AFF4 has revealed the structure of that complex (Figure 1), and demonstrated that the two components are unfolded until they bind one another (Leach et al, 2013). The authors argue that coupling protein folding and binding may facilitate the exchange of ligands between the proteins, while retaining high binding affinity and specificity. More generally, the flexible architecture of AFF4 (and related scaffolds) appears ideally suited for promoting dynamic protein–protein interactions, perhaps allowing the different elongation factors to be reeled in or reconfigured as transcription progresses.

The mechanisms by which super elongation complexes assemble and function are of general interest because these complexes also help regulate cellular gene expression at the level of transcriptional elongation (Luo et al., 2012). The coupling of multiple transcription factors into a single complex allows for signals such as HIV Tat:TAR or cellular DNA-binding proteins to trigger the rapid, synchronous activation of RNA polymerase complexes that have already been recruited, and which are poised to complete transcription once the brakes are removed.

Obtaining a detailed structural and mechanistic understanding of the entire Tat:TAR:pTEFb:AFF4 network is also of considerable interest for the development of HIV therapeutics. To this end, it will be important to understand precisely how the HIV Tat:TAR complex contacts pTEFb, particularly through the TAR RNA loop. Our knowledge of the structure of the Tat:TAR:cyclin T1 complex in a related virus that afflicts horses may offer some clues (Anand et al., 2008), but detailed analyses of the HIV system would help in developing therapeutically useful small molecules. Indeed, the pTEFb:AFF4 analyses already make it clear that the scaffold should be taken into account when screening for small molecules that inhibit the activation of Tat. Indeed, the structure itself inspires hope because the cyclin T1:AFF4 interface creates a pronounced Tat-binding groove that may be targetable.

Paradoxically, there is also interest in identifying small molecules that can *stimulate* HIV transcription. This is to facilitate immune recognition and destruction of the otherwise undetectable quiescent T cells that confound efforts to eradicate HIV by allowing the virus to remain invisible to the immune system (Mbonye and Karn, 2011). Hence, the quest to uncover fundamental biological principles and identify new therapeutic strategies should continue to fuel detailed structural and mechanistic studies of the elaborate mechanism of HIV transcriptional control.

## References

[bib1] Anand K Schulte A Vogel-Bachmayr K Scheffzek K Geyer M 2008 Structural insights into the cyclin T1-Tat-TAR RNA transcription activation complex from EIAV. Nat Struct Mol Biol 15:1287–92 doi: 10.1038/nsmb.1513 19029897

[bib2] Chou S Upton H Bao K Schulze-Gahmen U Samelson AJ He N 2013 HIV-1 Tat recruits transcription elongation factors dispersed along a flexible AFF4 scaffold. Proc Natl Acad Sci USA 110:E123–31 doi: 10.1073/pnas.1216971110 23251033 PMC3545800

[bib3] D'Orso I Jang GM Pastuszak AW Faust TB Quezada E Booth DS 2012 Transition step during assembly of HIV Tat:P-TEFb transcription complexes and transfer to TAR RNA. Mol Cell Biol 32:4780 doi: 10.1128/MCB.00206-12 23007159 PMC3497596

[bib4] He N Liu M Hsu J Xue Y Chou S Burlingame A 2010 HIV-1 Tat and host AFF4 recruit two transcription elongation factors into a bifunctional complex for coordinated activation of HIV-1 transcription. Mol Cell 38:428–38 doi: 10.1016/j.molcel.2010.04.013 20471948 PMC3085314

[bib5] Leach BI Kuntimaddi A Schmidt CR Cierpicki T Johnson SA Bushweller JH 2013 Leukemia fusion target AF9 is an intrinsically disordered transcriptional regulator that recruits multiple partners via coupled folding and binding. Structure 21:176–83 doi: 10.1016/j.str.2012.11.011 23260655 PMC3545106

[bib6] Luo Z Lin C Shilatifard A 2012 The super elongation complex (SEC) family in transcriptional control. Nat Rev Mol Cell Biol 13:543–7 doi: 10.1038/nrm3417 22895430

[bib7] Mbonye U Karn J 2011 Control of HIV latency by epigenetic and non-epigenetic mechanisms. Curr HIV Res 9:554–67 doi: 10.2174/157016211798998736 22211660 PMC3319922

[bib8] Ott M Geyer M Zhou Q 2011 The control of HIV transcription: keeping RNA polymerase II on track. Cell Host Microbe 10:426–35 doi: 10.1016/j.chom.2011.11.002 22100159 PMC3478145

[bib9] Razooky BS Weinberger LS 2011 Mapping the architecture of the HIV-1 Tat circuit: a decision-making circuit that lacks bistability and exploits stochastic noise. Methods 53:68–77 doi: 10.1016/j.ymeth.2010.12.006 21167940 PMC4096296

[bib10] Schulze-Gahmen U Upton H Birnbeg A Bauo K Chou S Krogan NJ 2013 The AFF4 scaffold binds human P-TEFb adjacent to HIV Tat. eLife 2:e00327 doi: 10.7554/eLife.00327 23471103 PMC3589825

[bib11] Sobhian B Laguette N Yatim A Nakamura M Levy Y Kiernan R 2010 HIV-1 Tat assembles a multifunctional transcription elongation complex and stably associates with the 7SK snRNP. Mol Cell 38:439–51 doi: 10.1016/j.molcel.2010.04.012 20471949 PMC3595998

[bib12] Tahirov TH Babayeva ND Varzavand K Cooper JJ Sedore SC Price DH 2010 Crystal structure of HIV-1 Tat complexed with human P-TEFb. Nature 465:747–51 doi: 10.1038/nature09131 20535204 PMC2885016

